# A retrospective case-control study of facial emotion recognition in male veterans with chronic schizophrenia and its correlation with interpersonal communication

**DOI:** 10.1186/s12888-022-04498-7

**Published:** 2023-01-18

**Authors:** Yu-Hong Wang, Xin-Fu Wang, Li-Da Shi, Xiao-Mei Xu, Li-Ning Wei, Shan-Shan Li, Xi-Po Li, Xin-Li Ma, Zhan-Min Li, Xin-Zhen Wei, Qian Wang, Ke-Qiang Wang

**Affiliations:** Department of Psychiatric rehabilitation, Hebei Province Veterans Hospital, No. 2396 Lianchi South Street, Baoding City, Baoding, 071000 China

**Keywords:** Veterans, Schizophrenia, Facial emotion recognition, Social skills, Interpersonal relationship, Social cognition, Rehabilitation

## Abstract

**Objective:**

To understand the facial emotion recognition of male veterans with chronic schizophrenia and the relationship between facial emotion recognition and interpersonal communication to provide a reference for designing social skills training programmes.

**Method:**

Fifty-six eligible male patients with chronic schizophrenia who were admitted to our hospital from October 2020 to April 2021 were selected, and 24 healthy people were selected as controls. Facial emotion recognition, social communication skills and self-perceived interpersonal disturbance were assessed using a facial emotion recognition stimulus manual, the Social Skills Checklist (SSC) and the Interpersonal Relationship Integrative Diagnostic Scale (IRIDS). Disease status was assessed using the Positive and Negative Syndrome Scale.

**Results:**

Both the control group and the patient group had the highest recognition accuracy for neutral faces. The recognition rate for neutral expression was higher in the control group than in the patient group (*p* = 0.008). The rate of neutral expressions identified as happiness was higher in the patient group than in the control group (*p* = 0.001). The identification of anger as happiness was higher in the control group than in the patient group (*p* = 0.026), and the pattern of misidentification was similar between the control group and the patient group. The accuracy of facial emotion recognition was negatively associated with the age of onset (*p* < 0.05). The recognition accuracy for happiness was negatively associated with negative symptoms, general pathological symptoms and total scale scores (*p* < 0.05). The total score for expression recognition was negatively associated with the negative symptom subscale scores (*p* < 0.05), and there was no correlation between expression recognition and positive symptoms (*p* > 0.05). The recognition accuracy for happiness was negatively correlated with the IRIDS conversation factor (*p* < 0.05). The recognition accuracy for happiness and anger and the total scores for facial emotion recognition were negatively correlated with the SSC subscale score and the total score (*p* < 0.05 and *p* < 0.01, respectively). The main influencing factors on facial emotion recognition were the SSC total score (*p* < 0.001) and the positive factor score (*p* = 0.039).

**Conclusion:**

Veterans with chronic schizophrenia have facial emotion recognition impairments affected by negative symptoms. There is a correlation between facial emotion recognition and interpersonal communication.

**Highlights:**

1. There are extensive facial expression recognition disorders in schizophrenia.

2. The pattern of misidentification was similar in both the control group and the patient group, with the tendency for happiness to be identified as a neutral emotion, anger as happiness, and fear as neutral emotion and anger.

3. Based on the comprehensive assessment of social cognitive impairment in patients with schizophrenia, prospective studies of standardised interventions are designed to provide support for clinical practice.

## Introduction

Social cognition is a multi-dimensional and multi-structural advanced cognitive function that includes understanding others’ psychological states, predicting others’ thoughts and judging others’ behaviours. Patients with schizophrenia have different degrees of social cognitive function impairment, which may be a stable quality defect that existed before the onset of the disease [1]. The main areas of social cognition research in schizophrenia are emotion perception, the theory of psychology and attributional style. Patients with schizophrenia have problems with emotion perception (recognising emotions expressed in various facial expressions or intonations) and facial emotion recognition function impairment [2, 3], and they spend less time examining facial features during emotion perception tasks [4].

Facial emotion recognition is a type of social cognitive function. Whether they can accurately identify facial emotions, recognise the moods and emotions of others and respond appropriately reflects the development of individual social ability to some extent. Therefore, the study of individual facial emotion recognition is of great importance.

Interpersonal communication has the function of transmitting information, communicating feelings and maintaining friendships. The basic form of communication is the expression of language and emotions. In the process of interpersonal interaction, whether a person can correctly understand another person’s language, identify their expression and interpret their intention will affect the outcome of their interpersonal communication. Social skills affect people with schizophrenia in terms of returning to and integrating with both their families and society. Improving social skills, and thus improving social functions and quality of life, are of great significance for both patients and their families; therefore, this has become one of the basic rehabilitation contents for patients with schizophrenia.

According to the actual situation in our hospital, we conducted a retrospective study of male veterans with schizophrenia and healthy people to analyse the characteristics of the defects in facial emotion recognition and the relationship with social skills and self-perceived interpersonal disturbance. This will help increase the awareness of patients’ interpersonal communications and provide a reference for designing social skills training programmes.

## Methods

### Participants

This was a retrospective case-control single-centre study. All the subjects in the patient group were inpatients from our hospital who were admitted between October 2020 and April 2021. 24 healthy people were selected as the control group. Enrolment criteria: Patients who met the diagnostic criteria of schizophrenia in the *International Classification of Diseases Tenth Edition* (ICD-10), were aged 25–65 years, were male, with a disease of more than 5 years, with an education level above primary school and with the ability to understand and communicate in Chinese. Exclusion criteria: Patients with a combined diagnosis of other diseases other than ICD-10 schizophrenia, with severe physical disease and/or hearing and visual dysfunctions.

This study was approved by the ethics committee of Hebei Rongjun Hospital (No. 2019005), and the patients and their family members provided signed informed consent.

### Test methods

For the self-evaluation tests, such as facial emotion recognition and interpersonal relationship integrative tests, three to five subjects were included in each session. To evaluate facial emotion recognition, each person had a stimulation manual, which they completed under the guidance of the testing physician. For the interpersonal relationship integrative test, the tester gave standardised guidance, and the subjects completed the test by themselves; after completion, it was checked and collected by the evaluating doctor. The social skills checklist (SSC) and the Positive and Negative Syndrome Scale (PANSS) were completed by the testing doctor. The testing tools used are described below.

**Fig. 1:**
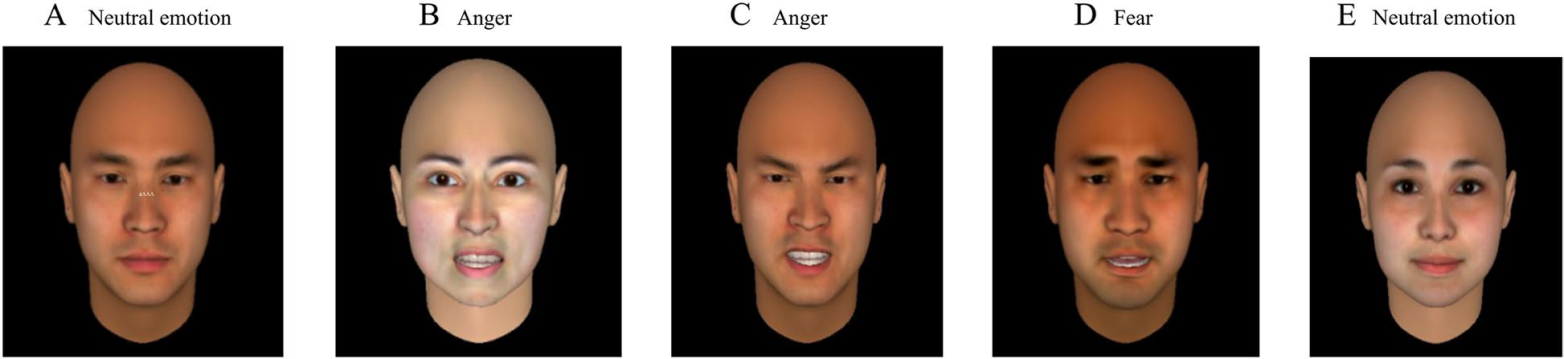
Facial emotion recognition stimulus manual

1. Facial emotion recognition stimulus manual: The stimulation manual used in the study was compiled by Zhang Xiangyang’s team in Beijing Huilongguan Hospital. The facial recognition stimulation images were computer-synthesised Chinese male and female faces showing emotions (Fig. 1), including neutral emotion, happiness, anger and fear. Each emotion had 16 faces out of a total of 64. The image sequence was divided into four parts, each containing 16 pieces, with four emotions randomly ordered in each part. After careful observation, the subjects gave their own opinion, scoring 1 point for a correct decision and 0 for an error.
2. Positive and Negative Symptom Scale [5]: As an evaluation tool for the status of the disease, the PANSS was designed and standardised for the assessment of different types and degrees of schizophrenia symptoms, with 30 entries. Of these, 7 are classified as positive, and the other 7 are negative. The remaining 16 are general psychopathology scales to assess the overall severity of schizophrenia (grades 1–7). The PANSS is widely used to measure psychotic symptoms and has good validity and reliability [6].
3. Social Skills Checklist [7]: The SSC is used by observers to assess the social communication skills of patients with mental disorders who are aged 18 years and above. There are 12 entries, divided into three aspects: conversation ability, relationship-building ability and conflict-handling ability. Each entry is scored from grades 0–4, and the higher the score, the more serious the damage. The test–re-test reliability of the Chinese version of the SSC is 0.93 ~ 0.99, the rater consistency is 0.92, and the internal consistency is 0.81 ~ 0.94 [8].
4. Interpersonal Relationship Integrative Diagnostic Scale (IRIDS) [9]: This is a self-rating scale to test the disturbance of interpersonal relationships. It contains 28 problems, divided into four aspects: conversation, communication, manner of dealing with people, and heterosexual friendships. The higher the score, the higher the degree of interpersonal relationship disturbance. The overall Cronbach’s α is 0.83, which showed that the scale has good reliability and validity.

### Data processing

All data were entered into a computer, and statistical processing was performed using Statistical Package for the Social Sciences (SPSS, version 19.0, IBM) software. Continuous data following a normal distribution or an approximately normal distribution with homogeneity of variance were described in terms of ± standard deviation, and comparisons between groups were analysed using the LSD method. Pearson’s correlation analysis was performed on the influencing factors, and a stepwise regression analysis was performed using the stepwise method. The variable entry criterion was set at 0.05, and the exclusion criterion was 0.1. The Chi-squared test was used for count data, and a two-sided value of *P* < 0.05 was used as the criterion for differences with statistical significance.

## Results

### Basic information of participants

A total of 56 patients completed the test, of which 49 were qualified. The patients were aged 54.02 ± 9.03 years. The age of onset was 21.07 ± 2.55 years, and the duration of education was 9.35 ± 4.00 years. The disease course was 33.02 ± 8.12 years. On the PANSS scale, the positive symptoms were 10.39 ± 4.34, the negative symptoms were 18.48 ± 8.64, and the general pathological symptoms were 27.61 ± 8.57, with a total score of 56.47 ± 18.36. The antipsychotic medication of the patients was converted to olanzapine at an equivalent of 15.59 ± 7.67 mg/day. The control group was from the general social population, and a total of 24 people completed the test. Their age was 54.13 ± 3.95 (45–60) years, with an education duration of 8.88 ± 2.25 years. There was no significant difference between the two groups in terms of age and duration of education (*p* > 0.05).

### Facial emotion recognition results

The overall identification accuracy of the control group and the patient group was 43.38 and 40.27%, respectively. The control group and the patient group had the highest accuracy for emotion recognition in terms of neutral emotion, reaching 55.99 and 47.63%, respectively, and the lowest fear recognition rate, at 32.26 and 33.59%, respectively.

A comparison of the identification rates between the control group and the patient group showed that the control group had higher rates of neutral expression recognition than the patient group, with statistically significant differences (*p* = 0.008). The rate of identifying neutral expressions as happiness was higher in the patient group than in the control group, with a statistically significant difference (*p* = 0.001). The rate of identifying anger as happiness was higher in the control group than in the patient group, with a statistically significant difference (*p* = 0.026). The rate of identifying anger as fear was higher in the patient group than in the control group (*p* = 0.057). Other identification accuracy and error rates did not differ between the two groups (*p* > 0.05) (see Table 1).

**Table 1:** Comparison of face emotion recognition results (%) between the control group and the patient group

The original face emotion	Neutral emotion (%)		Happiness (%)		Anger (%)		Fear (%)	
	the control group	the patient group	the control group	the patient group	the control group	the patient group	the control group	the patient group
Neutral emotion	55.99	47.63**	14.06	20.92**	17.71	18.16	12.24	13.29
Happiness	39.95	34.95	38.64	37.98	12.01	15.11	9.40	11.90
Anger	9.46	12.05	33.25	26.89*	43.54	41.99	14.51	19.07
Fear	23.68	25.03	19.47	17.90	21.58	23.48	35.26	33.59

*p < 0.05；**p < 0.01；

The within-group comparisons of the facial recognition results in the control group and the patient group showed statistically significant differences between the four outcomes (*p* < 0.001). Pairwise comparisons within the two groups showed similar patterns of misidentification in the control and patient groups, with happiness tending to be identified as a neutral emotion, anger toward happiness, and fear toward neutral emotion and anger (see Table 2).

**Table 2:** Within-group comparison of face recognition results (n) in the control group and the patient group

The original face emotion	the control group (n)				the patient group (n)			
	Neutral emotion	Happiness	Anger	Fear	Neutral emotion	Happiness	Anger	Fear
Neutral emotion	215^a^	54^b^	68^b^	47^b^	362^a^	159^b^	138^b^	101^c^
Happiness	153^a^	148^a^	46^b^	36^b^	266^a^	289^a^	115^b^	91^b^
Anger	33^a^	126^b^	165^c^	55^a^	91^a^	203^b^	317^c^	144^d^
Fear	90^a^	74^a^	82^a^	134^b^	193^a^	138^b^	181^a^	259^c^

^abcd^The same letter indicates no significant difference (*p* > 0.05), and different letters indicates statistically significant difference (*p* < 0.05)

The correlation analysis of expression recognition and the age of onset, disease course, age and duration of education, showed that the accuracy of facial emotion recognition was negatively correlated with the age of onset, with a statistical significance (*p* < 0.05), while it was negatively related with disease course, age and duration of education, without statistical significance (*p* > 0.05) (see Table 3).

**Table 3:** Correlation analysis of expression recognition and age of onset, disease course, age, and time of education

Items	Neutral emotion	Happiness	Anger	Fear	Total score
Age	−.128	−.202	−.107	−.072	−.260
Education	.074	−.147	−.162	−.012	−.101
Disease course	−.099	−.157	−.045	−.068	−.185
Age of onset	−.135	−.207	−.230	−.036	−.323*

*Correlation is significant at the 0.05 level (2-tailed)

### Facial recognition and psychiatric symptoms

A correlation analysis of facial recognition with the three subscales and the total PANSS was performed. The results showed that the recognition accuracy for happiness was negatively associated with negative symptoms, general pathology and the total score (*p* < 0.05), while the total expression recognition score was negatively associated with the negative symptom subscale score (*p* < 0.05). There was no correlation between expression recognition and positive symptoms (*p* > 0.05) (see Table 4).

**Table 4:** Correlation between face recognition and psychiatric symptoms

Items	Neutral emotion	Happiness	Anger	Fear	Total score
Positive symptom subscale	.070	−.018	.239	.227	.069
Negative symptom subscale	−.230	−.358^*^	.001	.119	−.362^*^
General pathological symptoms	−.073	−.461^**^	.029	.014	−.245
Total PANSS score	−.126	−.388^**^	.070	.009	−.222

*Correlation is significant at the 0.05 level (2-tailed)

**Correlation is significant at the 0.01 level (2-tailed)

### Facial recognition and interpersonal communication

A correlation analysis of the accuracy of facial emotion recognition was conducted using the conversation, communication, interpersonal behaviour, heterosexual communication and total scores of the IRIDS, and the conversation, relationship establishment, conflict resolution and total scores of the SSC scale. The results showed that emotion recognition accuracy was less correlated with IRIDS than with SSC, and only the recognition accuracy for happiness was negatively associated with the conversation factor of IRIDS (*p* < 0.05). The recognition accuracy for happiness was negatively associated with conversation ability, relationship building and the total SSC score (all *p* < 0.05). The recognition accuracy for anger and the total score of expression recognition were negatively correlated with the SSC subscale and the total score (all *p* < 0.05). Additionally, there was no correlation between the accurate recognition rate for neutral emotions and fear and the SSC scale (see Table 5).

**Table 5:** Results of the correlation analysis between face emotion and IRIDS and SSC

Items	Neutral emotion	Happiness	Anger	Fear	Total score
Conversation factor	−.158	−.323*	.012	−.055	−.263
Communication factor	−.176	−.213	.184	−.087	−.150
the way one gets along with people	−.160	−.281	.098	−.214	−.257
heterosexual communication	−.285	.025	.103	−.041	−.128
Total IRIDS score	−.225	−.237	.128	−.125	−.232
Conversation ability	−.201	−.465**	−.295*	−.032	−.526**
relationship-building	−.284	−.324*	−.355*	.005	−.511**
Conflict resolution	−.220	−.242	−.359*	−.025	−.454**
Total SSC score	−.249	−.398**	−.347*	−.020	−.543**

*Correlation is significant at the 0.05 level (2-tailed)

**Correlation is significant at the 0.01 level (2-tailed)

### Factors affecting facial recognition

The influencing factors were analysed further using face recognition as an independent variable. The results showed that the total SSC score (*t* = − 4.288, *p* < 0.001) and the positive factor score (*t* = 2.134, *p* = 0.039) were the main influencing factors, with R^2^ = 0.361.

## Discussion

### Facial emotion recognition results

According to the results of this study, both the control group and the schizophrenia patient group had emotion recognition disorders. The two groups were similar in terms of identification rate and error patterns. Both groups had the highest accuracy rate of neutral expression recognition and the lowest recognition rate of fear. Furthermore, both groups tended to identify happiness as neutral and anger as happiness. This result may be due to the same sociological basis of emotional recognition in healthy people and patients, which is formed during the process of socialisation. The patients maintained their original social cognitive characteristics even after disease onset.

Our studies have found differences between the two groups in the identification of neutral emotion and anger. The correct identification rate for neutral emotion in the patient group was lower than that of the control group, with the patient group tending to identify neutral emotions as happiness. The control group tended to identify anger as happiness, and the patient group tended to identify anger as fear. The facial emotion recognition process is often influenced by an individual’s previous social experience. While the control group understood the real thinking behind the emotion, the patient group remained at the superficial level of the face and was affected by thinking disorders. The difference in the recognition process may be the psychological factor of this difference. Yao et al. [10] showed that patients with first-episode schizophrenia had facial emotion recognition disorders before medication, and the injury of the frontal white-matter fibre may be the basis of pathophysiology. Therefore, more research is needed to further understand this difference.

The results of the current study showed that the overall recognition rate of facial emotions was negatively associated with the age of onset and had no correlation with disease course, age or duration of education. Neutral emotions, happiness, anger and fear were not correlated with disease course, age, age of onset or duration of education. Chen et al. [11] showed that male patients with schizophrenia were stable during the course of emotion recognition disorder. A meta-analysis by Kohler et al. [12] revealed that the age of onset was moderately associated with emotion perception. The later the age of onset, the greater the injury. However, the age of onset was not significantly associated with the course of the disease, which is a similar result to that of our study.

However, there are also inconsistent findings. Hofer et al.’s [13] studies on outpatients found that emotion recognition was positively associated with education and negatively associated with increasing age. Zhu et al. [2] showed that the accuracy of overall facial emotion recognition and angry faces were negatively correlated with the disease course of patients. In the present study, the patients were older and had a long disease course, but the results showed that the patients’ facial emotion recognition impairments did not worsen further with a prolonged disease course, suggesting that facial emotion recognition impairments in patients with schizophrenia may be a functional impairment that is independent of the disease.

Facial emotion recognition impairments in schizophrenia occur from the first episode of the disease [14] and are also present in patients’ children and siblings [15, 16]. As the disease develops, facial emotion recognition disorders show stable features [17]. Moreover, the scores of aphasia recognition tests and the recognition scores for sadness, panic, fear and anger in the eye area of emotion tests in patients with schizophrenia were lower than those in healthy controls [18]. All these studies suggest that deficits in social cognitive function may be schizophrenia-specific impairments, indicating that they may be genetic endophenotypes of schizophrenia.

### Facial emotional recognition and psychiatric symptoms

This study showed that the identification of happiness was correlated with psychiatric symptoms and negatively associated with negative symptoms, general pathology and total scale scores, while it had no correlation with neutral emotion, anger or fear. The total score for facial recognition showed a negative correlation with negative symptoms and no correlation with positive symptoms. This result is not consistent with those of previous studies [2, 12], and it may be related to the higher age, longer disease course and more prominent negative symptoms in this study. The patient still has positive residual symptoms, but the impact on the patient was weaker than their negative symptoms and general pathological symptoms. The more severe the psychiatric symptoms in the study, the lower the recognition rate for happiness.

Patients may be affected by their moods when recognising facial emotions. In this group, the patients had dull emotions, anhedonia and less happiness experience. The identification results also reflected the mood of the patients to some extent, which may be the reason that it significantly affected only the identification of happiness. The results differ regarding the correlation between facial emotion recognition and psychiatric symptoms. However, these results still suggest that when designing social skills training programmes, we need to increase certain elements to improve negative symptoms and initial motivation, apply cognitive and behavioural therapy techniques promptly, correct the core beliefs, reduce pathological thinking interference, improve social cognition levels and reduce the negative effects of psychiatric symptoms.

### Facial emotion recognition and interpersonal communication

The results showed that the correlation between the IRIDS total score, each factor score and emotion recognition accuracy (except happiness and conversation) was not statistically significant. However, the correlation between the SSC total score, the score for each factor, and the happiness and anger recognition rates (except happiness and conflict resolution) was statistically significant. Happiness recognition was negatively associated with conversation factors, showing higher happiness recognition to be correlated with low conversation disturbance. They were good at using the appropriate form of language to exchange ideas, but conversation without trouble cannot cover up or offset issues with communication, interpersonal behaviour and heterosexual communication. In general, the correlation between the accuracy of facial emotion recognition and the subjects’ self-perception of interpersonal disturbance was not obvious, reflecting that the patients did not recognise interpersonal disturbances, indicating a defect in their interpersonal self-perceptions. Facial emotion recognition was significantly associated with social skills, and it affected social skills. The higher the accuracy of the identification, the less impaired the social skills. The results also showed that the identification results for happiness and anger affected interpersonal interactions more than neutral emotions and fear. In interpersonal communication, whether or not we can correctly identify happiness and anger has a great impact on social communication skills. In the process of social communication, emotional recognition is an important link. Only with the correct emotional interpretation can there be proper emotional expression, and language communication can occur smoothly within the correct emotional interaction. In interpersonal communication practice, the importance of emotional communication exceeds even that of language communication; this reminds us to consider facial emotion recognition and interpersonal self-perceptions when designing interpersonal communication training.

This study has the following limitations: The stimulus images used in the study were computer-synthesised Chinese facial expressions, without difficulty classification, an internal consistency test or a validity test. In addition, the test tool was in a static image format, but real expressions are dynamic, continuous and contain emotional interaction and communication. Whether or not there are differences in the recognition results of static images and dynamic expressions, we need to develop new testing tools for in-depth research. Previous studies have shown differences between different genders, with women with schizophrenia performing better than men in terms of facial emotion recognition [19, 20]. There are significant gender differences in the pattern of error rates in male and female patients [21], although our study subjects were of a single gender. The lack of female subjects, the small sample size of the patients and the lack of differential analysis of the images of the emotional faces of different genders are the shortcomings of this paper. All these problems need to be supplemented and improved in subsequent studies. The social cognition used in the study also includes the theory of psychology, and corresponding test tools have been developed [22] to assess the severity of cognitive impairment in the psychological theory of schizophrenia. In the future, prospective studies of standardised interventions can be designed based on the comprehensive assessment of social cognitive disorder in schizophrenia to accumulate evidence-based medical proof and provide further support for clinical practice.

In conclusion, veterans with chronic schizophrenia have impaired facial emotion recognition, the accuracy of which is negatively associated with the age of onset and is affected by negative symptoms.

## Data Availability

All data generated or analyzed during this study are included in this article.
